# Quality Assessment of Fruits and Vegetables Based on Spatially Resolved Spectroscopy: A Review

**DOI:** 10.3390/foods11091198

**Published:** 2022-04-20

**Authors:** Wan Si, Jie Xiong, Yuping Huang, Xuesong Jiang, Dong Hu

**Affiliations:** 1 College of Mechanical and Electronic Engineering, Nanjing Forestry University, Nanjing 210037, China; sw@njfu.edu.cn (W.S.); xiongjie0612@163.com (J.X.); xsjiang@126.com (X.J.); 2 College of Optical, Mechanical and Electrical Engineering, Zhejiang A&F University, Hangzhou 311300, China; hudong538338@zju.edu.cn

**Keywords:** spatially resolved spectroscopy, optical properties, fruits and vegetables, quality assessment

## Abstract

Damage occurs easily and is difficult to find inside fruits and vegetables during transportation or storage, which not only brings losses to fruit and vegetable distributors, but also reduces the satisfaction of consumers. Spatially resolved spectroscopy (SRS) is able to detect the quality attributes of fruits and vegetables at different depths, which is of great significance to the quality classification and defect detection of horticultural products. This paper is aimed at reviewing the applications of spatially resolved spectroscopy for measuring the quality attributes of fruits and vegetables in detail. The principle of light transfer in biological tissues, diffusion approximation theory and methodologies are introduced, and different configuration designs for spatially resolved spectroscopy are compared and analyzed. Besides, spatially resolved spectroscopy applications based on two aspects for assessing the quality of fruits and vegetables are summarized. Finally, the problems encountered in previous studies are discussed, and future development trends are presented. It can be concluded that spatially resolved spectroscopy demonstrates great application potential in the field of fruit and vegetable quality attribute evaluation. However, due to the limitation of equipment configurations and data processing speed, the application of spatially resolved spectroscopy in real-time online detection is still a challenge.

## 1. Introduction

Nowadays, optical imaging and spectroscopy technologies are widely used in the quality and safety assessment of fruits and vegetables. The quality of fruits and vegetables has also attracted more attention, especially under the serious circumstances of the global COVID-19 pandemic, which is forcing people to rethink their lifestyles and diets. This will undoubtedly introduce many opportunities and challenges into the quality evaluation of fruits and vegetables. From the initial focus on external detection to the current research on the internal quality evaluation of fruits and vegetables [1], many achievements have been made following the decades of development of optical imaging and spectroscopy technology, coupled with many newly emerging problems.

Optical detection technology is mainly based on the principle of interaction between light and biological tissues. Absorption and scattering effects will occur when the light source illuminates the biological tissue. The absorption is mainly related to chemical composition, while the scattering is often thought to be connected with the physical structure of biological tissues. Therefore, the internal quality of biological materials changes with their physical structure and chemical composition, resulting in changes in the optical properties of biological tissues [2,3,4]. Thus, optical properties derived from mathematical models and chemometrics are exploited in visible and near infrared (VIS/NIR) spectroscopy to realize the quality evaluation of biological materials [5,6,7,8]. Norris [9] first applied NIR spectroscopy in the agricultural field to measure moisture in grains. In the past two decades, a great number of studies have focused on the quality parameter evaluation of fruits and vegetables, focusing on firmness [10,11], bruises [12,13], maturity [14,15], soluble solids [16,17] and acidity [18]. However, conventional VIS/NIR spectroscopy selects a single point or specific area to measure the quality parameters, and cannot provide spatial information of the test sample [19]. Moreover, direct methods are required to measure the total amount of light reflected from or transmitted through biological tissues. On the one hand, the absorption and scattering effects cannot be distinguished in this way. On the other hand, the direct measurement approach has inherent shortcomings because the measured value of reflectance or transmittance is external or phenomenological and is closely related to the instrument type, the sensing mode, and the light source/detection probe’s settings [20], which limits the accuracy of the quality measurement and evaluation of fruits and vegetables.

In order to remove this limitation, researchers have begun to study the optical absorption and scattering characteristics of biological tissues separately, and many new technologies have emerged, such as time-domain technology [21,22], frequency-domain technology [23,24], spatial frequency-domain technology [25,26] and spatially resolved technology [27,28]. Spatially resolved technology estimates the absorption and scattering characteristics by measuring the reflectivity at different distances from a light source of constant intensity and employing an inversion algorithm combined with the diffusion approximation equation [29,30]. This approach was first developed by Reynolds et al. [31] to understand the mechanism of light propagation in turbid media. Later, Langerhoic [32] and Marquet et al. [33] reported that spatially resolved technology can be used to determine the optical properties of biological tissues. Spatially resolved spectroscopy (SRS) offers information at different depths of biological tissues. A transmission path, often “banana-shaped”, is formed when photons pass through the biological tissues [34]. Therefore, surface layer information can be obtained with a shorter distance between source and detector, and deeper layer information can be derived when using a greater source-detector distance. Consequently, spatially resolved spectroscopy is helpful to assess the condition and characteristics of heterogeneous-structure tissues at different depths. This technology has been used in biomedical research for the non-invasive diagnosis and monitoring of abnormal tissues in the human body [35,36,37,38] and studies on optical properties and variety identification of wood [39,40]. In recent years, it has also been widely applied in the detection of internal defects (bruising, mealiness, etc.) and the quality attributes (maturity, firmness, acidity, etc.) of agricultural products.

Therefore, in the following, the principle of light transmission in biological tissues and the diffusion approximation theory are explained. Then, different potential ranges, advantages, and problems of the spatially resolved spectroscopy are described in relation to different instrument configurations. The application for SRS in fruit and vegetable quality detection is also reviewed from two aspects of optical properties and spatially resolved (SR) spectra. Finally, the challenge and problems for previous studies are discussed and analyzed, and then future development trends are given.

## 2. Principle and Methodology

### 2.1. Light Transfer in Biological Tissues

To understand the principle of spatially resolved spectroscopic technology when used in fruit and vegetable quality detection, we must first understand how light propagates in and interacts with biological tissues, and how to apply this propagation law to the actual application of fruit and vegetable quality inspection.

**Figure 1:**
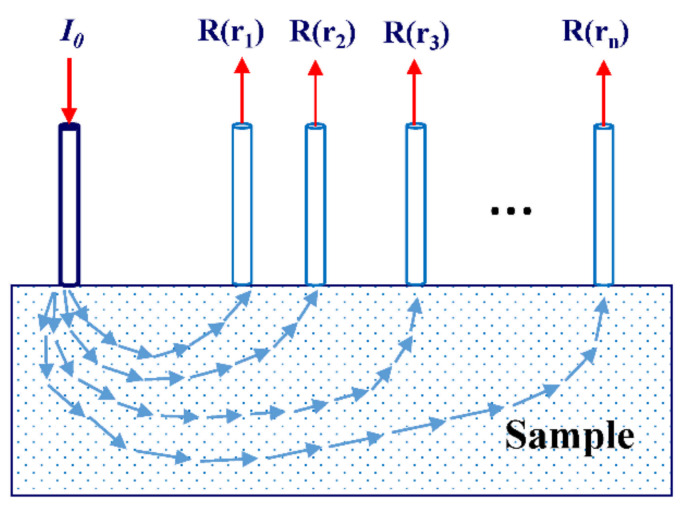
The configuration of single-fiber scanning and the “banana-shape” path of light propagation in the sample.

From the microscopic point of view, biological tissue is composed of a large number of tiny cells. They are mainly composed of the nucleus, cytoplasm, and various organelles [41]. Each of them contains different physical structures and chemical components, which means that their optical properties are also very different. Therefore, it is a very complex matter to study the interaction between light and biological tissue on the micro-scale. However, we can simplify the light propagation model to see that it is mainly composed of absorption and scattering [42]. When a photon enters a chaotic medium and meets the particles in the medium, it may be directly absorbed, and the light energy is then transformed into other forms of energy (such as heat energy). This process is called absorption. Scattering may also occur, which changes the propagation direction, leading to an encounter with another particle in the medium. The above absorption or scattering process repeats until the photon is finally absorbed or leaves the medium [43]. Spatially resolved spectra contain information about samples at different depths, as the path of photon transmission is usually “banana-shape” in biological tissues [34], as shown in Figure 1.

The light absorption characteristics of biological tissues mainly depend on its chemical components, such as pigment, water, carbohydrate, etc. (for fruit and vegetable products). We usually use the absorption coefficient (*μ_a_*) to describe the ability of biological tissues to absorb light. The light scattering characteristics of biological tissue are mainly related to its physical structure, such as cell density, cell volume, and so on. Similarly, we will use the scattering coefficient (*μ_s_*) to describe the light scattering ability of biological tissues [44].

However, light scattering is inhomogeneous, proliferating in all directions in biological tissues. The anisotropy coefficient (*g*) is introduced to measure this inhomogeneity. Combined with the scattering coefficient (*μ_s_*), the reduced scattering coefficient (*μ′_s_*) is obtained to reduce the variables of the transmission model (*μ′_s_* = *μ_s_*(1 − *g*)) [45].

### 2.2. Diffusion Approximation Theory

The propagation of light in biological tissues can be described by the radiation transfer equation (or the Boltzmann equation) [46,47]. However, it is expressed in integral and differential forms containing multiple independent variables, which are difficult to solve both numerically and analytically. Therefore, the model simplification method can be obtained under certain conditions.

For biological materials dominated by scattering effects (*μ′_s_* >> *μ_a_*), the radiation transfer equation can be simplified to a diffusion approximation equation. Two analytic solutions commonly used at present were respectively proposed by Farrell and Patterson [48], and Kienle and Patterson [49]. By extrapolating boundary conditions, Farrell and Patterson [48] obtained an approximate analytical solution for the diffusion of a single layer of tissue as:

(1) $$R(r)=\frac{a^{\prime }}{4\pi }\left[z_{0}\left(\mu_{eff}^{\prime }+\frac{1}{r_{1}}\right)\frac{\exp\left(-\mu_{eff}^{\prime }r_{1}\right)}{r_{1}^{2}}+\left(z_{0}+2z_{b}\right)\left(\mu_{eff}^{\prime }+\frac{1}{r_{2}}\right)\frac{\exp\left(-\mu_{eff}^{\prime }r_{2}\right)}{r_{2}^{2}}\right]$$

where r is source-detector distance, $r_{1}={\left(z_{0}^{2}+r\right)}^{1/2}$ is the distance from the detector to the actual light source, $r_{2}={\left({\left(z_{0}+2z_{b}\right)}^{2}+r^{2}\right)}^{1/2}$ is the distance from the detector to the mirror light source, $\mu_{eff}^{\prime }={\left[3\mu_{a}\left(\mu_{a}+\mu_{s}^{\prime }\right)\right]}^{1/2}$ is the effective attenuation coefficient, $z_{0}={\left(\mu_{t}^{\prime }\right)}^{-1}={\left(\mu_{a}+\mu_{s}^{\prime }\right)}^{-1}$, $z_{b}=2AD$, $D={\left(3\mu_{t}^{\prime }\right)}^{-1}$, $A=\frac{1+R_{f}}{1-R_{f}}$ is the reflection coefficient inside the tissue, $R_{f}$ is determined by the refractive index of the tissue.

Kienle and Patterson [49] introduced radiant energy flow rate into this model, and expressed diffuse reflectance as a linear combination of radiant energy flow rate and luminous flux, which can help effectively reduce errors and more accurately describe the propagation characteristics of light in biological tissues. The radiant energy flow rate can be expressed as follows:

(2) $$\Phi (r)=\frac{1}{4\pi D}\left(\frac{\exp\left(-\mu_{eff}r_{1}\right)}{r_{1}}-\frac{\exp\left(-\mu_{eff}r_{2}\right)}{r_{2}}\right)$$

The diffusion approximation equation can be transformed as follows:

(3) $$\begin{array}{c} R(r)=\frac{C_{1}}{4\pi D}\left[\frac{\exp\left(-\mu_{eff}r_{1}\right)}{r_{1}}-\frac{\exp\left(-\mu_{eff}r_{2}\right)}{r_{2}}\right]+\\ \frac{C_{2}}{4\pi }\left[\frac{1}{\mu_{t}^{\prime }}\left(\mu_{eff}+\frac{1}{r_{1}}\right)\frac{\exp\left(-\mu_{eff}r_{1}\right)}{r_{1}^{2}}+\left(\frac{1}{\mu_{t}^{\prime }}+2z_{b}\right)\left(\mu_{eff}+\frac{1}{r_{2}}\right)\frac{\exp\left(-\mu_{eff}r_{2}\right)}{r_{2}^{2}}\right] \end{array}$$

where $C_{1}=\frac{1}{4\pi }\int_{2\pi }\left[1-R_{\mathrm{fres}\;}(\theta )\cos\theta d\omega \right]$ and $C_{2}=\frac{3}{4\pi }\int_{2\pi }\left[1-R_{\mathrm{fres}\;}(\theta )\cos^{2}\theta d\omega \right]$ are the coefficients related to the different refractive indexes of the medium. For fruit and vegetable products, the refractive index n is 1.35, $C_{1}$ = 0.1277, $C_{2}$ = 0.3269 [31].

### 2.3. Methodology

Before conducting experiment, the spatially resolved measurement system is first built, including a point light source or narrow collimated beam with constant intensity, detectors with different source-detector distances and imaging spectrograph [29]. Optical fiber arrays and hyperspectral line-scan imaging are two common configurations in the spatially resolved measurement system [50,51,52,53], which will be described in detail in the following section. The system needs to be calibrated before spectral data acquisition. The main objectives of calibration include linear calibration, spectral response calibration and optical property calibration. Generally, different calibration procedures are adopted according to different experimental objectives [45,54]. The spectral data acquisition should be performed after finishing all calibration procedures. The initial value of the obtained spectral data is detector counts, which should be calibrated by white and black spectra to obtain relative spectra [55]. The process above is called a data preparation stage.

**Figure 2:**
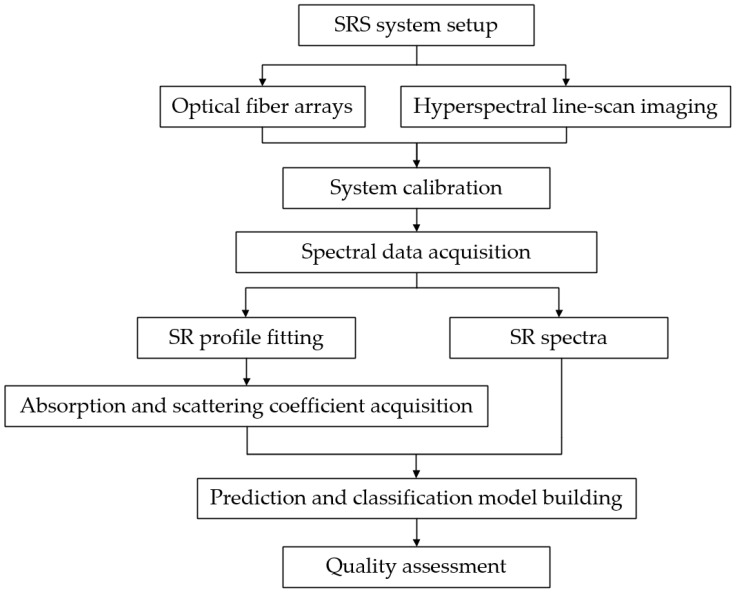
Flowchart of complete experimental procedures based on the spatially resolved spectroscopy measurement system. (SRS = spatially resolved spectroscopy; SR = spatially resolved).

In the stage of data processing, spatially resolved spectra could be applied in different analysis fields. On the one hand, the spatially resolved spectra is capable of assessing the quality attribute of biological materials by establishing the classification or prediction models [56]; on the other hand, optical absorption and scattering coefficients were extracted from spatially resolved spectra by an inverse algorithm, and the quality attribute could be evaluated by optical property parameters [57]. Figure 2 shows the flowchart of complete experimental procedures based on the spatially resolved spectroscopy measurement system.

## 3. Instruments

Depending on the detection equipment used, spatially resolved technology is generally divided into two detection methods: one based on optical fiber arrays, and the other on hyperspectral line-scan imaging.

### 3.1. Optical Fiber Arrays

There are two types of fiber array, i.e., the single-fiber scanning configuration and the multi-fiber array configuration. The single-fiber scanning configuration generally involves only two optical fibers, one for transmitting optical signals and the other for detecting optical signals, as shown in Figure 1. During the measurement process, the position of the optical fiber acting as the light source is unchanged, and optical signals at different positions are received by moving the detection optical fiber [58], so as to obtain a spatially resolved diffuse reflection spectrum. This detection method has the advantages of its simple structure and convenience of operation. However, due to the different surface curvatures of fruit and vegetable products, frequent changes in position will inevitably bring about measurement errors, and the spectral data must be read every time the position is moved, which is time-consuming [29]. In addition, the long-term exposure of biological tissues (muscle tissue or pulp tissue) to the air may cause pollution [58,59], and the optical properties may thus change, rendering it impossible to access the original characteristics of the biological tissues.

**Figure 3:**
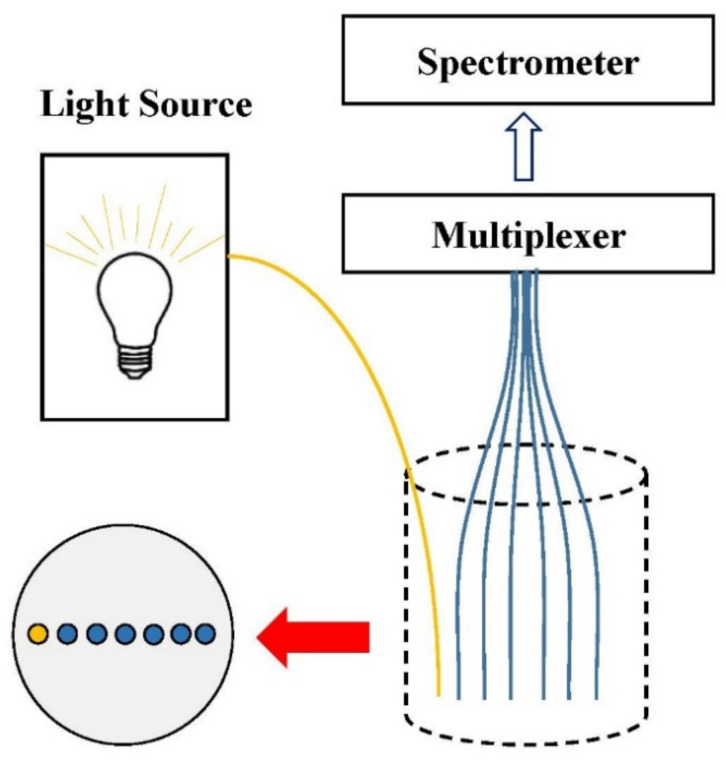
The configuration of the multi-fiber array based on a multiplexer.

Therefore, the researchers have developed a detection device based on a multi-fiber array, which couples an illumination fiber and multiple detection fibers with a multiplexer fixed at a certain distance, and then obtains the reflected signal through a spectrometer [29]. As shown in Figure 3, the illumination and detection fibers were arranged in a line to receive the signals returned from the sample [60]. The spatially resolved spectra could be obtained from detection fibers which do not need to move during the whole experimental process using a visible and near-infrared spectrometer coupled with a multiplexer. However, the spectral data obtained via the multiplexer in this SRS configuration still needs to be read sequentially in order to obtain optical signals.

**Figure 4:**
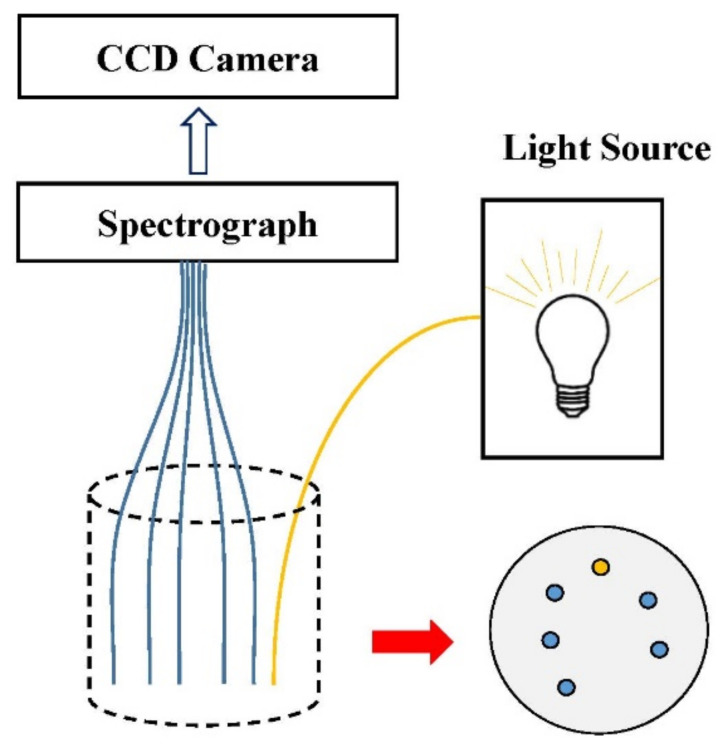
The configuration of the ring arrays based on an imaging spectrograph. (CCD = charge-coupled device).

In order to solve this problem, a fiber optic probe based on a ring array has been developed. The illumination fiber and the detection fiber are distributed in a ring, and the other end is coupled to the imaging spectrograph in the charge-coupled device (CCD) camera, as shown in Figure 4. Trong et al. [61] developed this configuration for non-destructive testing of Braeburn apples, which enabled the simultaneous collection of spectral signals when using different source-detector distances (maximum 1.2 mm), which greatly improved the rate of spectral data reception. Dried apple slices and optical liquid phantoms were investigated based on this configuration for acquiring scattering and absorption properties of samples [62]. The fiber-array configuration is often applied on liquid foods, dried fruits slices and leafy vegetables due to the good contact between the probe and the sample surfaces [29,45,63].

**Figure 5:**
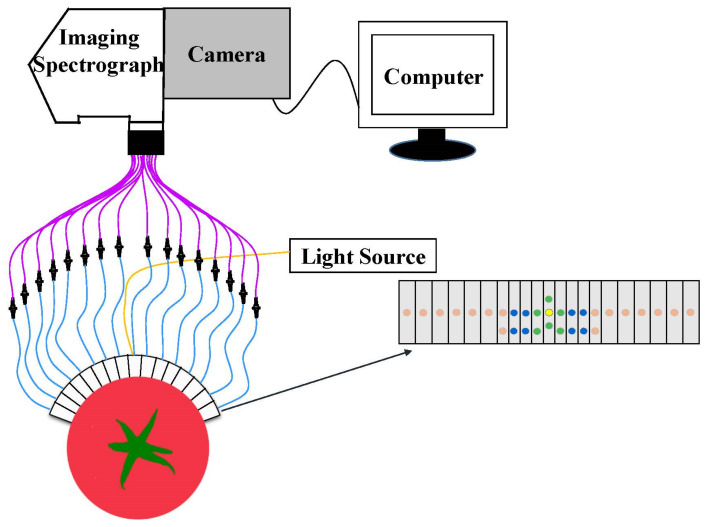
The configuration of the multi-channel curved array of SRS.

However, as it is limited by the size and shape of the probe, this configuration is only suitable for samples with a small source-detector distance and a flat surface [64,65]. Especially when used for fruit and vegetable products, the detection probe cannot fully contact the sample surface, which will cause large measurement errors. Based on this, Huang et al. [66] developed a multi-channel curved array detection system, as shown in Figure 5. The detection probe can maintain good contact with the sample surface, and the source-detector distance can be increased to 36 mm. The horizontal extension of the detection position can enable one to obtain better-quality information pertaining to the sample, and improve the accuracy and reliability of the detection experiment. This configuration was used to evaluate various quality attributes of apples and tomatoes, and good experimental results were obtained due to the reasonable design of the detection system [63,67,68,69,70].

### 3.2. Hyperspectral Line-Scan Imaging

**Figure 6:**
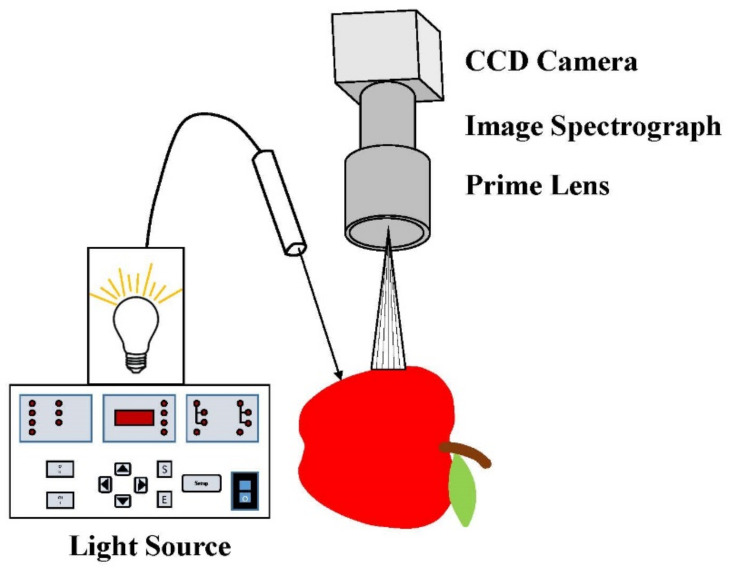
The configuration of hyperspectral line-scan imaging.

At present, the most commonly used measurement method within spatially resolved technology is hyperspectral line-scan imaging [42,71], as shown in Figure 6. Optical fibers are not used to transmit optical signals in the measurement method of line-scan imaging, but a CCD camera that is set above the sample surface is integrated into the imaging system to realize non-contact measurement. The light source used in the imaging system does not need to keep close contact with the measured sample, but can maintain a certain distance from the sample’s surface [72]. In order to obtain the spectral information for multiple wavelength bands of the sample at the same time, a line-scan imaging spectrograph is installed at the front end of the CCD camera to decompose light. When collecting images, one-dimensional spatial information and complete spectral information related to the samples can be obtained after one repetition of line scanning [73]. In addition, to avoid saturation in the collected image resulting from the camera directly scanning the incident point of the light source, the scanning line usually needs to deviate by a certain distance relative to the incident point of the light source [74]. The method of hyperspectral line-scan imaging can realize non-contact measurement, avoiding the contact error caused by the optical fiber measurement method, and has a large spatial range and high spatial resolution. At present, it is widely applied in the evaluation of fruit and vegetable quality attributes, such as apple [75], peach [76], tomato [77], pear and kiwi fruit [44]. It can measure multiple wavelengths at the same time, and is suitable for the assessment of the quality characteristics of agricultural products and food [78,79].

Table 1 shows different SRS configurations applied by researchers, which will be described in the following chapters. Compared to the method based on optical fiber arrays, configurations of hyperspectral line-scanning are used more frequently in studies of quality attributes of different fruit and vegetable products. Moreover, regarding the selection of the spectral range, the spectrum in the 500–1000 nm range is the first choice to conduct research, probably because the spectral data in this range contains information relating to VIS and NIR bands, which generates less noise and is helpful to improve the accuracy of the research. In summary, the number and arrangement of optical fibers are two key factors for the SRS configuration of fiber-array. The number of optical fibers determines the efficiency of the spectrometer to receive data, whereas the arrangement of optical fibers determines the quality of signals received from the sample. The multi-channel detection system developed by Huang et al. [45] is integrated with a flexible probe fixed 30 detection fibers, which can be kept in good contact with the sample surface. The configuration of fiber-array is more suitable for research or investigation in the laboratory with small error and high reliability and precision, but it is not suitable for real-time online application. A hyperspectral line-scan imaging system is usually composed of a light source, zoom lens, spectrograph and CCD camera, which benefits from its non-contact measurement method [29,80,81]. The quality of the acquired signal varies with different light sources, source-detector distances, imaging modes and calibration procedures [20].

## 4. Optical Properties Application

In the beginning, spatially resolved spectroscopy was mostly used in the field of medical diagnosis, to carry out the inspection of diseased or abnormal tissues of the human body by analyzing the propagation characteristics of light in biological tissues. Later, it was used to evaluate the quality attributes of fruits and vegetables. The specific steps are as follows: firstly, based on diffusion theory and the reverse algorithm, the spectral data are fitted to obtain the absorption coefficient (*μ_a_*) and reduced scattering coefficient (*μ′_s_*) values related to the optical properties of fruits and vegetables, and they are then combined with mathematical models based on the *μ_a_* and *μ′_s_* values to realize the prediction of quality attributes of fruits and vegetables. The study of optical properties has generally been aimed at quality attribute assessment, defect detection, and layered structure analysis.

### 4.1. Absorption and Scattering Coefficients Analysis

The absorption and scattering spectra reflect the propagation of light in the tissue, which could provide a preliminary understanding of the internal and external quality properties of the sample. By observing the absorption coefficient and reduced scattering coefficient spectra of the sample, the absorption and scattering characteristics of experimental objects with different wavelength bands and conditions can be obtained, which is of great use in the analysis of fruit and vegetable quality.

With the increase in the number of wavelengths, the absorption coefficient spectrum shows a fluctuating trend as a whole. For different fruits and vegetables, although the absorption spectrum of each sample is different, the overall profile shape is roughly the same. For example, Cen et al. [93] illustrated the optical absorption and scattering spectra to analyze the optical properties of ‘Red Star’ peaches at different levels of maturity, the absorption spectra of peaches at three different levels of maturity (light red, red, dark red) show two prominent absorption peaks near 675 nm and 970 nm, caused by the uptake of chlorophyll and water into the fruit tissue, respectively. During the ripening process of a peach, the content of chlorophyll decreases, while the content of anthocyanin increases and become the dominant pigment. When evaluating the optical properties of liquid foods (juice) and various fruits and vegetables (apple, peach, pear, kiwifruit, plum, cucumber, zucchini squash, and tomato), Qin et al. obtained the conclusion that the main pigments affect the absorption spectra of samples and the researchers also classify tomatoes into different ripeness stages correctly using the ratio of the absorption coefficient at 675 nm (for chlorophyll) to that at 535 nm (for anthocyanin) [44,97]. The same results were also derived from the previous studies on exploring the relationship between absorption spectra and internal chemical components of fruits and vegetables [54,82,87,94].

While for the spectral profile of the reduced scattering coefficient for peach fruit presented in Cen’s study [93], it is relatively flat and has fewer features. On the whole, it shows a downward trend with the increase in the number of wavelengths. This trend is consistent with Mie’s scattering theory, which says that scattering is caused by spherical particles and is strongly wavelength-dependent [98]. As the peach matures with hardness gradually softens, the reduced scattering coefficient decreases accordingly. For soft fruits, a sharp drop in the *μ′_s_* value may invalidate the scattering-dominant assumption, and the diffusion model will thus give erroneous *μ_a_* and *μ′_s_* spectra [93]. Lu studied the optical properties of bruised apples [88] and mechanically damaged pickled cucumbers [96], and found that after the damage occurred, the scattering properties of the samples decreased to a greater extent than the absorption properties. Therefore, an optical system that enhances the measurement of scattering characteristics will be more suitable for the detection of damaged samples, which is of great significance for the detection and identification of internally defective fruits and vegetables. In Hu’s study, a double-fiber-optical-probe system was established and developed for optical absorption and scattering property measurement of 32 pear samples with three different measured surfaces in the spectral region of 500–1000 nm [85].

It can be seen that the internal chemical composition or surface texture characteristics of fruits and vegetables can be distinguished by a simple trend chart of absorption or scattering coefficients. However, for the prediction or classification of quality attributes, optical parameters need to be combined with relevant mathematical models to realize a more comprehensive assessment of fruit and vegetable quality.

### 4.2. Maturity and Quality Assessment

Consumer satisfaction with fruits and vegetables is commonly related to their maturity. The maturity of fruits and vegetables generally affects their quality and market price. It is important to study the changes taking place in optical properties at different maturity stages for the quality detection and classification of fruits and vegetables. Qin and Lu [44] classified tomatoes by observing the differences in the absorption spectra of tomatoes at three different maturity stages (green, pink, and red), and using the ratio of the absorption coefficient at 675 nm to that at 535 nm. Zhu et al. [77] studied the changes in the light absorption and scattering characteristics of tomatoes during ripening, and established a classification model of tomato ripening using light absorption and scattering spectra. The results show that the light absorption spectra and scattering spectra, and especially their combination, are useful for the classification of tomato maturity. Huang et al. [45] developed a new multi-channel hyperspectral imaging probe to evaluate the optical properties of fruits and vegetables and achieved the accurate detection of the optical properties of tomatoes at different maturity levels (green, turning, pink, red) in the band of 550–1350 nm. During the process of tomato ripening from green to red, their surface gradually becomes softer, along with a gradual decrease in *μ′_s_* value, which will provide a reliable evaluation method for the prediction and classification of the quality attributes of fruits and vegetables in the future.

For the maturity assessment of fruits and vegetables, surface color discrimination is a common approach, as described above. However, the firmness and soluble solids content (SSC) are thought to be internal quality characteristics closely related to the maturity of garden products. Therefore, it is necessary to explore the relationship between quality attributes and optical characteristic parameters. In experimental research, standard reference values are often set by means of physical and chemical experiments. Firmness values can be measured by the Magness Taylor test, while SSC values are usually determined by the Brix meter. As mentioned above, the absorption and scattering coefficient values are obtained by fitting the spectral data, and they are then correlated with the standard value of the quality attribute to judge the accuracy of the prediction model.

Qin et al. [86] measured the absorption and reduced scattering coefficients of Golden Delicious apples using spatially resolved spectroscopy, and correlated them with fruit firmness and SSC. The results show that both *μ_a_* and *μ′_s_* were related to fruit firmness and SSC, and the same conclusion was also achieved by Qin and Lu [44] (tomato), Cen et al. [93] (Red star peach), and Trong et al. [82] (Braeburn apple). In addition, Cen et al. [93] demonstrated that firmness and SSC were also correlated with the skin and flesh color parameters of peaches, and the prediction results were better than those derived from the acoustic and impact measurement experiments. In addition to using a single optical characteristic parameter (*μ_a_* or *μ′_s_*) for quality attribute prediction, the combination of the absorption coefficient and the reduced scattering coefficient can improve the prediction results [76]. This method was applied by Qin et al. [86] and Cen et al. [87] for the prediction of apple firmness and SSC, and the model prediction accuracy was unsurprisingly improved. Qin et al. [86] also improved the efficiency of model prediction by screening the optimal wavelength; however, for fruit firmness and SSC, the number of wavelengths required for prediction and the region of wavelength distribution were all different. The wavelengths of the predicted SSC were mainly distributed in the short-wave near-infrared region, probably because the light absorption in this region was relatively flat and small. Firmness and SSC are usually two common quality attributes, which normally appeared in the research of fruit and vegetable quality evaluation, since firmness reflects the physical structure characteristics, while SSC is related to the chemical composition of the sample. In general, the combination of absorption and scattering coefficients usually can improve the prediction results of firmness and SSC, which is worthy of further investigation in the future. The above studies have clearly demonstrated the potential use of SRS method in the non-destructive quality assessment of fruits and vegetables.

### 4.3. Defect Detection

Defect detection is focused on the application of classification on damaged or diseased fruits and vegetables. Some fruits are likely to be damaged during picking or postharvest transportation, such as apples and peaches. It is necessary to analyze the optical properties of these fruits to judge the relationship between damage and fruit defect detection. Using SRS technology based on hyperspectral imaging, Lu et al. [88] studied the changes in absorption coefficient (*μ_a_*) and reduced scattering coefficient (*μ′_s_*) of normal and damaged tissues of two different varieties of apples (Golden Delicious and Red Delicious). They found that bruising would change the *μ_a_*, but the change patterns of two different varieties of apples were not consistent. In addition, the scattering coefficients (*μ_s_*) of normal apples were much higher than those of bruised apples. Therefore, an optical system for measuring enhanced scattering features will be more useful for damaging detection. Similarly, they investigated the optical absorption and scattering characteristics of normal and internally defective pickled cucumbers, and found that the influence of the total mechanical damage on the scattering properties is greater than that on the absorption properties [96]. The results show that enhanced scattering measurement can effectively detect defects via the optical property evaluation of pickled cucumber, which confirms their previous conclusion. For the problem of peach bruising, Sun et al. [95] measured the optical property coefficients of peaches at different levels of maturity from 550 to 1000 nm. It was found that both maturity and bruising led to a decrease in *μ′_s_* and an increase in *μ_a_*. Moreover, internal damage to the peach tissue can be observed via the optical properties before the appearance of symptoms observable by the naked eye, which confirms the potential utility of fruit optical property assessment compared with manual selection.

In the process of postharvest transportation and storage, fruits are prone to become diseased as a result of fungal infection, which will bring about unnecessary economic losses in the fruit industry. Sun et al. [94] measured the absorption coefficient and reduced scattering coefficient of peaches inoculated with fungi using spatially resolved technology, and found that the value of *μ′_s_* decreased with the aggravation of peach disease. Moreover, *μ_a_* and *μ′_s_* showed the highest correlation with chlorophyll content in the first 72 h after inoculation. This means that the optical absorption and scattering properties can provide effective information useful for early disease detection in peaches.

Mealiness is an internal disorder without any obvious external symptoms but affects the taste of apples. Three classification models were used to sort mealy and non-mealy apples with spatially-resolved scattering images at 650 and 980 nm in Kaveh’s study [92]. An overall classification accuracy of 74% was obtained for the artificial neural network (ANN) model based on a combination of all effective optical parameters acquired at both wavelengths. More highly correlated wavelengths should be added for better classification performances in the future. Table 2 shows main studies of various fruits and vegetables and models used in quality attribute evaluation.

### 4.4. Layered and Microscopic Structure Application

Most fruits and vegetables are multiple-layer structures. The depth of light penetration into fruit and vegetable tissues is much greater than the thickness of the peel, and ranges from 2 to 4 mm [99] for apples and 0.97 to 6.52 mm [44] for fruit and vegetable products. This means that part of the light (excluding the light scattered and absorbed by the peel) will pass through the peel tissue, and then continue to be absorbed or scattered by the pulp tissue [101]. The same model has often been used for peel and pulp tissue in previous studies on the optical properties of fruits and vegetables. However, the optical properties of peel and pulp tissue may be quite different. Therefore, different mathematical models should be adopted for peel and pulp tissues, respectively.

Askoura et al. [100] found that a peeled apple diffused light more effectively than an apple with skin in an investigation on light propagation through apple tissue structures. When using spatially resolved spectroscopy to study optical properties, ignoring the fruits’ different tissue structures in order to simplify the experimental model will inevitably bring about errors in the experimental results. Therefore, it is very important to regard fruit as composed of two different layers of peel and pulp. Cen and Lu [102] developed an inverse algorithm and a two-layer diffusion model. The optical properties of the top layer of the model sample were determined: the error of the absorption coefficient was less than 23.0%, and the error of the reduced scattering coefficient was less than 18.4%. However, the error in the lower layer’s optical parameters became very large, and it was difficult to measure the optical properties of the sub-layer, even when the optical parameters and thickness of the top layer were known. Later, a sequence estimation method for estimating the optical properties of two-layer media with spatially resolved diffuse reflectance was proposed by Wang et al. [103]. They found that the optical properties of the top layer could be determined with less than 10% error when using the semi-infinite diffusion model, and when the thickness of the top layer was larger than 2 mfp (mean free path). Furthermore, the optical properties of the bottom layer were estimated to less than 10% error with the two-layer diffusion model when the thickness of the top layer was less than 16 mfp. Although the use of a two-layer diffusion model could theoretically improve experimental predictions, there are still considerable challenges in implementing this approach in real samples.

Great majority of studies have been conducted on various quality attributes of fruits and vegetables from a macroscopic perspective. Cen et al. made a preliminary exploration on the relationship between the changes of optical properties and the microstructure of fruits [75]. The tissues of ‘Golden Delicious’ and ‘Granny Smith’ apples were analyzed by confocal laser scanning microscopy to obtain the microstructure parameters. Linear regression analysis showed that light absorption and scattering parameters were positively correlated with cell area and equivalent diameter. The relationship between optical properties and tissue microstructure of ‘Baifeng’ and ‘Xiahui 8’ peaches during storage has also been investigated in Ma’s research (single integrating sphere system) [104]. It suggests that optical properties can also be used to study the changes of microstructure of fruits during ripening and post-harvest storage.

## 5. Spatially Resolved Spectra Application

Spatially resolved spectroscopy is based on spectral data instead of optical property parameters, and is coupled with a selected mathematical model to directly predict the quality attributes, or to carry out a classification of fruits and vegetables. As Table 3 shows, although there are few papers in this field, the limited research still shows that the quality evaluation method based on SR spectra data has considerable reliability and application potential.

### 5.1. Quality Attribute Prediction

Bruising is also a common cause of losses to orchardists during the post-harvest transportation and storage of fruits. To quantify the bruise susceptibility of fruits, Zhu et al. [89] extracted the hyperspectral scattering characteristics of Golden Delicious apples in the wavelength range of 500–1000 nm using the average relative reflectance spectrum. Based on the partial least squares (PLS) model, the damage sensitivity of an apple after bruising can be predicted and analyzed, and good results can be obtained. The prediction correlation coefficient R_P_ = 0.848–0.919. This study shows that hyperspectral scattering can be used to evaluate the bruise sensitivity of apples, which will inform the post-harvest treatment of fruit.

Peng and Lu [71] proposed 10 different forms of modified Lorentz distribution functions to fit the spectral scattering profiles of the firmness and SSC of Golden Delicious apples in the wavelength range of 450–1000 nm. Each function showed superior fit with the data, with an average correlation coefficient greater than 0.995. Huang et al. [83] studied the prediction of the tomato soluble solids content and acidity (pH) based on spatially resolved spectroscopy and traditional VIS/NIR spectroscopy. Individual SR spectra or their combinations were developed, combined with PLS modeling. The results show that the combination with SR spectra generally led to more consistent and better prediction results for SSC and pH compared with a single SR spectrum. The same conclusion was got as before [63]. SR spectra enabled better pH prediction than VIS/NIR and NIR spectra. In the same year, another study [70] was undertaken on the firmness of tomatoes, and similar conclusions were obtained, which proves the broad application prospects of the spectra combination method based on spatially resolved spectroscopy for the quality assessment of fruit and vegetable products. Ma et al. [64] developed a multi-fiber-based SRS measurement system to simultaneously evaluate the firmness and SSC of apples. The determination coefficients of prediction were 0.92 for SSC and 0.87 for firmness. The goal of the rapid and precise measurement of apple firmness and SSC was achieved, showing the possibility of integrating the SRS method into the design of low-cost and portable devices in the field of quality attribute online monitoring.

### 5.2. Quality Attributes Classification

Mealiness is a symptom of physiological fruit disorder, characterized by the abnormal softness and the lack of free juice in the fruit. Huang and Lu [90] acquired spectral scattering profiles between 600 and 1000 nm using hyperspectral imaging system. Apples were classified based on mealiness with the PLS model. The overall classification accuracy of two categories of apples (“non-mealiness” and “mealiness”) was between 74.6% and 86.7% (the accuracy when applied to three and four categories decreased in turn). The classification accuracy was higher (>92%) after a long apple mealiness treatment, indicating that the hyperspectral scattering technology can effectively detect severely mealy apples, but is less useful for less mealy apples. Mendoza et al. [91] applied VIS/NIR spectroscopy and spatially resolved spectroscopy to classify three different varieties of apples, proving that the spatially resolved spectroscopic technology has the potential to classify and grade apples online through firmness and SSC.

Huang et al. [69] applied a self-developed multi-channel hyperspectral imaging system to distinguish different ripening stages of tomatoes. In total, 15 spatially resolved spectra were used for modeling based on partial least squares discriminant analysis (PLSDA) and support vector machine discriminant analysis (SVMDA), respectively. The results show that the best classification spectrum was not the same for different prediction models, and the prediction accuracy of SVMDA was higher than that of PLSDA. Based on different source-detector distance and SVMDA model, better classification results with total classification accuracy of 98.3% were obtained in the study of tomato maturity assessment [67]. They also proved that the potential applicability of spatially resolved spectroscopy for tomato color discrimination was greater than that of single-point VIS/NIR spectroscopy [84]. Later, research was conducted on the identification of apple varieties in three wavebands (550–780 nm, 780–1650 nm, and 550–1650 nm) based on the method of spectra combination [68]. The classification accuracy of the three spectra all reached 100%, but in the visible light range, the method of spectra combination could not improve the classification accuracy (compared to individual spectra classification). This shows that although the spectra combination method did not lead to a good classification accuracy in the range of individual bands, it still showed considerable application potential in the variety identification and maturity classification of fruits and vegetables.

## 6. Discussion

Currently, with the increase in human consumption levels and the changes in consumption acceptance, more attention is being paid to the issue of healthy diet habits, especially related to fruits and vegetables. In recent years, research and surveys based on spatially resolved spectroscopic technology have become prevalent, providing an effective and reliable method for the evaluation of the quality attributes of fruits and vegetables [20,74].

The optical fiber probe-based SRS method is simple in configuration, easy to operate, and can obtain quality information at different depths in the sample [29]. Great results have been achieved in fruit firmness and SSC measurement, even with the simplest algorithm. However, when using optical fiber probes for experimental research, special attention should be paid to the settings of the experimental platform and the configuration of the detection fiber. The instrument configuration needs to be specially set for fruits and vegetables with different surface curvatures. The SRS method based on hyperspectral line-scan imaging can achieve long-distance and non-contact measurement, avoiding the pollution caused by the contact between the sample and the lens, and experimental data could be obtained from a wider spectral range [72]. Often, superb model prediction and classification performances can be obtained in experimental research, and the accuracy and reliability of the experimental results can thus be guaranteed. The light beam and source-detector distance are two key factors in the configuration of the experimental instruments [20]. Cen and Lu reported that the optimal light beam should be of Gaussian type with the diameter of less than 1 mm, and the optimal minimum and maximum source-detector distance should be 1.5 mm and 10–20 mean free paths, respectively [55]. However, the above experimental results are based on liquid model samples. In view of the different optical properties for each fruit and vegetable, the optimal beam and source-detector distance may be various. Therefore, investigation needs to be implemented on the suitable configuration of different fruits and vegetables. Most fruits and vegetables are heterogeneous in structure and have irregular shapes, which makes the accurate measurement of optical properties more complicated. In the configuring of experimental instruments in the future, attention should be paid to the development of specific detection equipment and calibration procedure for the heterogeneous structures and irregular shapes of fruits and vegetables, which is a great challenge, but is imperative [97].

The SRS method also has limitations in fruit and vegetable quality evaluation [38]. Since the use of SRS is based on the diffusion approximation theory, which is only an approximate solution to the radiation transmission equation, it is only suitable for samples whose scattering effects are much greater than their absorption effects. Support vector regression (SVR) was introduced to simulate the relationship between diffuse reflectance and optical properties for removing limitations in numerical calculation of diffusion approximation equation [60]. In addition, Farrell and Patterson [48] studied the propagation law of light beams in semi-infinite media, which is not always applicable to horticultural products with large surface curvatures. The currently available optical properties measurement technology is still prone to produce errors in the process of data acquisition and the optical parameter inverse algorithm extraction, which also brings about errors in the prediction model, and prevent its full utilization in fruit and vegetable maturity and quality evaluation. The determination of the optical properties of a sample is a complex problem, which needs to be properly approached via pretreatment program setting and inverse algorithm construction. Data noise should be removed before fitting the absorption and scattering spectral profiles [20]. Logarithmic transformation and relative weighting methods give more reliable estimations of the optical parameters [57]. Therefore, the standardized processing of data acquisition and the selection of a reasonable inverse algorithm are necessary to reduce errors.

The selection of different mathematical models has a significant impact on the results of prediction or classification. At present, most of the models used in fruit and vegetable quality detection are PLS and SVM models. Neural network or deep learning algorithms based on spatially resolved spectroscopy have not been reported. Therefore, appropriate mathematical models should be developed for different fruits and vegetables, rather than using the same mathematical model. In addition, compared with the average reflectance method alone, the extraction of spectral data combined with image feature analysis can significantly improve the prediction results of the quality attributes of fruits and vegetables [75]. Extracting the wavelengths with high correlation is especially useful to reduce the spectral data and improve the detection speed. Integrated imaging modes (reflectance and transmittance, as well as reflectance and fluorescence) may be a major trend in future experimental research [105]. With the advancements of software and hardware, and the improvement of data processing speed of imaging equipment, SRS configuration based on hyperspectral line-scan imaging can be applied for real-time online detection in the future.

## 7. Conclusions

Considerable progress in research on spatially resolved spectroscopy has been made in the past 20 years, and it has demonstrated the great application potential in the field of fruit and vegetable quality attribute detection. This paper reviews the applications of spatially resolved spectroscopy in the quality evaluation of fruits and vegetables in detail. Applications on the prediction and classification of fruit and vegetable quality attributes are widely developed based on optical properties. SRS shows great potential in physical and chemical property evaluation, defect detection and tissue structure analysis. There are few studies on the applications of the quality evaluation directly based on spatially resolved spectra for fruits and vegetables. However, for directly spatially resolved spectra assessment, the data process is simpler than that for extracting optical properties, which reduces the possibility of introducing errors. Overall, the internal and external quality characteristics of fruits and vegetables can be detected simultaneously via spatially resolved spectroscopy. However, there are still great challenges in improving the detection speed and accuracy. Therefore, the comprehensive utilization of various processing means, including the design of instrument configuration, data preprocessing, the selection of the specific wavelength and model, and the combination with other imaging methods, can expand the SRS applications.

## Figures and Tables

**Table 1:** SRS configurations of optical fiber arrays and hyperspectral line-scan imaging with different wavelength regions.

Configurations	Products	Wavelength (nm)	References
OFA ^1^	Apple	400–1000	[82]
		750	[65]
		550–1650	[68]
		600–1100	[64]
		500–1000	[62]
	Tomato	550–1650	[45,54,63,66,67,69,70,83,84]
	Pear	500–1000	[85]
HLI ^2^	Apple	500–1000	[44,75,86,87,88,89]
		450–1000	[71]
		600–1000	[90]
		450–1050	[91]
		650, 980	[92]
	Tomato	500–1000	[44]
		500–950	[77]
	Peach	500–1000	[44]
		515–1000	[76,93]
		550–1000	[94,95]
	Cucumber	700–1000	[96]
		500–1000	[44]
	Pear	500–1000	[44]
	Plum		
	Kiwifruit		
	Zucchini squash		
	Juice	500–1000	[97]

^1^ OFA = optical fiber arrays. ^2^ HLI = hyperspectral line-scan imaging.

**Table 2:** Post-harvest quality assessment by optical properties of fruits and vegetables.

Products	Quality Attributes	Main Studies	Models	References
Apple	Firmness, SSC	Prediction for apple firmness and SSC.	PLS ^1^	[82]
			MLR ^2^	[86]
			PLS	[87]
	Mealiness	Investigate the possibility of non-destructive apple mealiness classification.	PCR ^3^, PLS, ANN ^4^	[92]
	Bruising	Knowledge of the spectral absorption and scattering properties of normal and bruised apple tissue.	—	[88]
	Tissue structure	Research on light penetration properties of ‘Jonagold’ apple tissue.	—	[99]
		Investigation on light propagation through apple tissue structures.	—	[100]
		Optical properties–microstructure–texture relationships of dried apple slices.	—	[62]
		Quantify the relationship of optical properties with structural and mechanical properties in apple tissues.	LRA ^5^	[75]
Peach	Firmness, SSC	Prediction of firmness and SSC for ‘Red Star’ peaches.	PLS LS-SVM ^6^	[93]
	Fungal infection	Determine the relationships of the optical parameters with structural and biochemical parameters during quality deterioration.	PCCA ^7^	[94]
	Bruising	Measure the optical coefficients of peaches after bruising at different maturity levels and detect bruises.	SVM ^8^	[95]
Tomato	Maturity	Measure the absorption and scattering coefficients of tomato fruit at four maturity stages.	—	[45,54]
		Ripeness evaluation and classification of ‘Sun Bright’ tomato.	PLS-DA ^9^	[77]
	Firmness, SSC	Prediction for tomato firmness and SSC by optical property parameters.	PLS	[66]
Cucumber	Defect	Measure the optical absorption and scattering properties of normal and internally defective pickling cucumbers.	—	[96]
Pear	Optical property	Optical property measurement of pear samples with a double-fiber-optical-probe system.	—	[85]
Juice	Optical property	Measure the absorption and scattering properties of turbid food materials.	LRA	[97]
Various products	Optical property	Optical property measurement for the samples of apple, peach, pear, kiwifruit, plum, cucumber, zucchini squash, and tomato. Classification of tomato at three ripeness stages.	—	[44]

^1^ PLS = Partial least square. ^2^ MLR = Multiple linear regression. ^3^ PCR = Principal component regression. ^4^ ANN = Artificial neural network. ^5^ LRA = Linear regression analysis. ^6^ LS-SVM = Least square support vector machine. ^7^ PCCA = Pearson correlation coefficient analysis. ^8^ SVM = support vector machine. ^9^ PLS-DA = Partial least square discriminant analysis.

**Table 3:** Post-harvest quality assessment by spatially resolved spectra of fruits and vegetables.

Products	Quality Attributes	Main Studies	Models	References
Apple	Firmness, SSC	Simultaneous evaluation of SSC and firmness in apple with a multifiber-based SRS measurement system.	PLS	[64]
		Evaluate and compare different mathematical models for describing the hyperspectral scattering profiles in order to select an optimal model for predicting firmness and SSC of apples.	MLR	[71]
	Mealiness	Detection and classification of mealy apples for investigating the potential of hyperspectral scattering technique.	PLS	[90]
	Bruising	Evaluate the changes of optical properties in tomatoes during ripening and develop classification models for grading the ripeness of tomatoes.	PLS-DA	[77]
	Variety classification	Classification of apple varieties based on individual spectra and spectral combination.	PLS-DA	[68]
		Sort three varieties of apple into two quality grades based on firmness, SSC, or both firmness and SSC.	LDA ^1^	[91]
Tomato	Maturity	Recognition for tomato surface color and internal color by SRS and conventional single point VIS/NIR spectroscopy.	PLS-DA	[84]
		Evaluate tomato maturity in different layers by using a newly developed spatially resolved spectroscopic system.	SVM-DA ^2^	[67]
		Tomato maturity classification based on different models and source-detector distance.	PLS-DA SVM-DA	[69]
	Firmness, SSC/pH	Quality assessment (SSC and pH) of tomatoes with single-point spectra and SR spectra using a newly developed SRS system.	PLS	[83]
		Quality evaluation of tomato fruit based on individual spectra and spectral combination with different source-detector distance.	PLS	[63]
		Determine optimal prediction models for the firmness parameters with individual SR spectra and their combinations.	PLS	[70]

^1^ LDA = Linear discriminant analysis. ^2^ SVMDA = Support vector machine discriminant analysis.

## Data Availability

No new data were created or analyzed in this study. Data sharing is not applicable to this article.
